# An Overview of Current Knowledge of Deadly CoVs and Their Interface with Innate Immunity

**DOI:** 10.3390/v13040560

**Published:** 2021-03-26

**Authors:** Yamei Zhang, Siobhan Gargan, Yongxu Lu, Nigel J. Stevenson

**Affiliations:** 1 Viral Immunology Group, School of Biochemistry and Immunology, Trinity Biomedical Sciences Institute, Trinity College Dublin, D02 R590 Dublin, Ireland; YZHANG6@tcd.ie (Y.Z.); GARGANSI@tcd.ie (S.G.); 2 Department of Pathology, University of Cambridge, Cambridge CB2 1QP, UK; yl581@cam.ac.uk; 3 Viral Immunology Group, Royal College of Surgeons in Ireland—Medical University of Bahrain, Adliya 15503, Bahrain

**Keywords:** viral structure, epidemiology, transmission, viral life cycle, innate immunity, treatment

## Abstract

Coronaviruses are a large family of zoonotic RNA viruses, whose infection can lead to mild or lethal respiratory tract disease. Severe Acute Respiratory Syndrome-Coronavirus-1 (SARS-CoV-1) first emerged in Guangdong, China in 2002 and spread to 29 countries, infecting 8089 individuals and causing 774 deaths. In 2012, Middle East Respiratory Syndrome-Coronavirus (MERS-CoV) emerged in Saudi Arabia and has spread to 27 countries, with a mortality rate of ~34%. In 2019, SARS-CoV-2 emerged and has spread to 220 countries, infecting over 100,000,000 people and causing more than 2,000,000 deaths to date. These three human coronaviruses cause diseases of varying severity. Most people develop mild, common cold-like symptoms, while some develop acute respiratory distress syndrome (ARDS). The success of all viruses, including coronaviruses, relies on their evolved abilities to evade and modulate the host anti-viral and pro-inflammatory immune responses. However, we still do not fully understand the transmission, phylogeny, epidemiology, and pathogenesis of MERS-CoV and SARS-CoV-1 and -2. Despite the rapid application of a range of therapies for SARS-CoV-2, such as convalescent plasma, remdesivir, hydroxychloroquine and type I interferon, no fully effective treatment has been determined. Remarkably, COVID-19 vaccine research and development have produced several offerings that are now been administered worldwide. Here, we summarise an up-to-date understanding of epidemiology, immunomodulation and ongoing anti-viral and immunosuppressive treatment strategies. Indeed, understanding the interplay between coronaviruses and the anti-viral immune response is crucial to identifying novel targets for therapeutic intervention, which may even prove invaluable for the control of future emerging coronavirus.

## 1. Human CoVs

Coronaviruses (CoVs) are positive-sense, enveloped RNA viruses. They are in the order *Nidovirale*, which contains four genera—alpha, beta, gamma and delta (α, β, γ and δ) [1]. So far, seven human coronaviruses (HCoV) have been identified and are known to cause human respiratory tract illness. Of note, most HCoVs originate from bat CoVs [2]. There are four HCoVs which primarily affect children, the elderly, and immunocompromised patients, of which HCoV-NL63 and HCoV-229E are alphacoronaviruses, and HCoV-HKU1 and HCoV-OC43 are betacoronaviruses [1,3,4,5]. These four HCoVs infections cause less severe “common cold” symptoms, including fever, sore throat, cough and bronchitis; while the remaining three HCoVs, Severe Acute Respiratory Syndrome (SARS)-CoV-1, Middle East respiratory syndrome coronavirus (MERS-CoV) and SARS-CoV-2, are more pathogenic and cause pneumonia and acute respiratory distress syndrome (ARDS) and are associated with a considerably high mortality rate. The recently emerged SARS-CoV-2 is more contagious, but less deadly than SARS-CoV-1 and MERS-CoV [6].

## 2. SARS-CoV-1

In November 2002 a patient presented with atypical pneumonia in Guangdong China. The causative agent was later identified as SARS-CoV-1. The virus is thought to have been transmitted from palm civets to humans at a food market in China [7]. The virus eventually infected 8,089 people, across 29 countries and caused 774 deaths (a mortality rate of 9.6% [8]) (Table 1). The clinical symptoms of SARS-CoV-1 infection include fever, cough, dyspnoea and respiratory failure [9]. Several therapies were used to treat patients infected with SARS-CoV-1, including ribavirin, corticosteroids and type I Interferon, however, there was no convincing evidence that they helped recovery [10]. The measures put in place to curtail the spread of the virus, such as travel restrictions and patient isolation, were successful and the virus was declared to be “contained” by the World Health Organization (WHO) in July 2003 [11].

## 3. MERS-CoV

MERS-CoV emerged in 2012. Similar to SARS-CoV-1, it spread from animals to humans. MERS-CoV is also highly infectious, with individuals being infected after short exposure times [28]. The virus was first identified in the Kingdom of Saudi Arabia (KSA), in a patient who died of progressive respiratory and renal failure. Laboratory analysis revealed it was caused by a pathogenic CoV that had not previously been reported in humans [29]. This novel virus initially caused an outbreak in the Middle East region, with a second major focus point emerging in 2015, in the Republic of Korea, which was thought to be spread by individuals travelling from Qatar, KSA and the United Arab Emirates (UAE) [30]. By the end of January 2021, there have been 2566 reported cases of MERS-CoV across 27 countries, and 881 have resulted in death (34.4% mortality). Even though MERS-CoV appears to have been curtailed, there were still 47 new cases reported by the WHO during last 12 months, highlighting the need for continued awareness and research to develop an understanding of this virus, with a view to developing curative medicines and a vaccine [21] (Table 1). 

## 4. SARS-CoV-2

**Figure 1:**
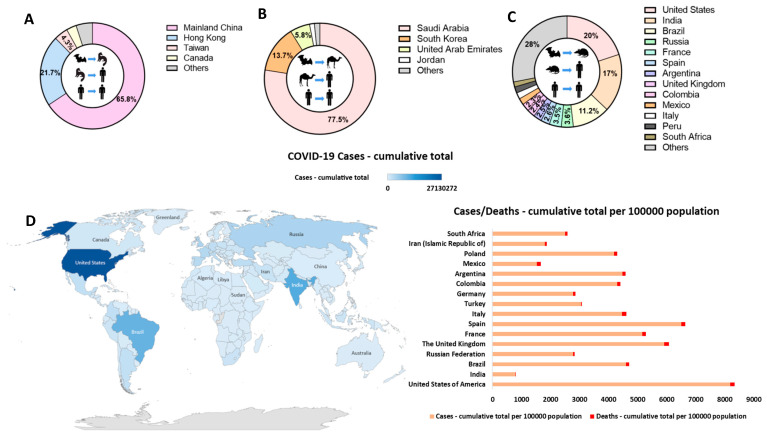
The transmission patterns and countries with the highest proportion of coronavirus diagnoses. Transmission patterns for (**A**) SARS-CoV-1, (**B**) MERS-CoV and (**C**) SARS-CoV-2 and countries with the largest proportions of confirmed cases are shown separately. (**D**) The COVID-19 cases and deaths—cumulative total per 100,000 population for countries with the largest proportions of confirmed cases. (The datasets analysed in this figure are available on the WHO website WHO|World Health Organization).

In December 2019, an outbreak of pneumonia-like disease occurred in Wuhan, China. The causative agent of this outbreak was later found to be a novel CoV, subsequently named SARS-CoV-2. The number of cases increased exponentially before March 2020, with over 80,000 cases and over 3000 deaths reported in China [31]. Although SARS-CoV-2 shares high sequence similarity with SARS-CoV-1, the basic reproductive number of SARS-CoV-2 has been estimated to be 2.68, this is higher than that of SARS-CoV-1, which was lower than 2 [32]. The virus spread rapidly across the globe and was declared a pandemic by the WHO on 11 March 2020. By the 10 March 2021, a staggering 117,764,619 confirmed cases and 2,613,747 deaths, in 215 areas worldwide, had been reported. The early SARS-CoV-2 cases were all thought to be linked to a seafood market in China, which also sold wild animals, again suggesting a zoonotic transmission of the virus [33] (Figure 1). SARS-CoV-2 causes COronaVIrus Disease (COVID-19), with symptoms including fever, sore throat, muscle aches, fatigue, dyspnoea and loss of taste [34]. While figures continue to change, COVID-19 is thought to have a mortality rate of 0.8–10.8% [35], but individuals with co-morbidities [36] and the elderly are at higher risk [37]. SARS-CoV-2 continues to circulate in our global population with vigour, causing unprecedented global lockdown of societies worldwide.

The seriousness of CoV infection has propelled the need for therapeutic cures and vaccines to the forefront of clinical research. Indeed, the zoonotic route of infection reveals the ease at which CoVs can “jump” between species and highlights the likelihood of future zoonotic transmission. The presence of animal CoV reservoirs increases the risk of novel strains emerging. Upon infection, the innate immune system detects the presence of CoVs and initiates signalling cascades, resulting in the downstream activation of anti-viral and pro-inflammatory responses. However, CoVs have evolved to attenuate anti-viral responses while also triggering a pro-inflammatory, immunopathogenic response. This review discusses the CoV life cycle, their interplay with immune signalling pathways and the current research and development of anti-viral drugs.

## 5. Name, Classification and Viral Structure

**Figure 2:**
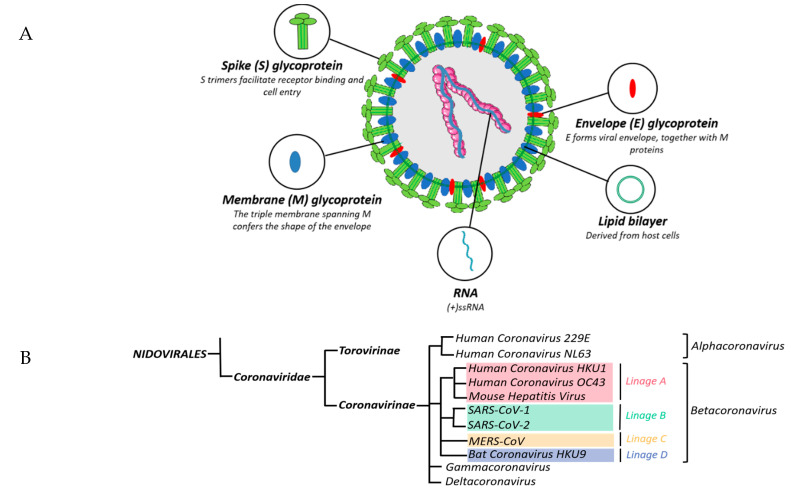

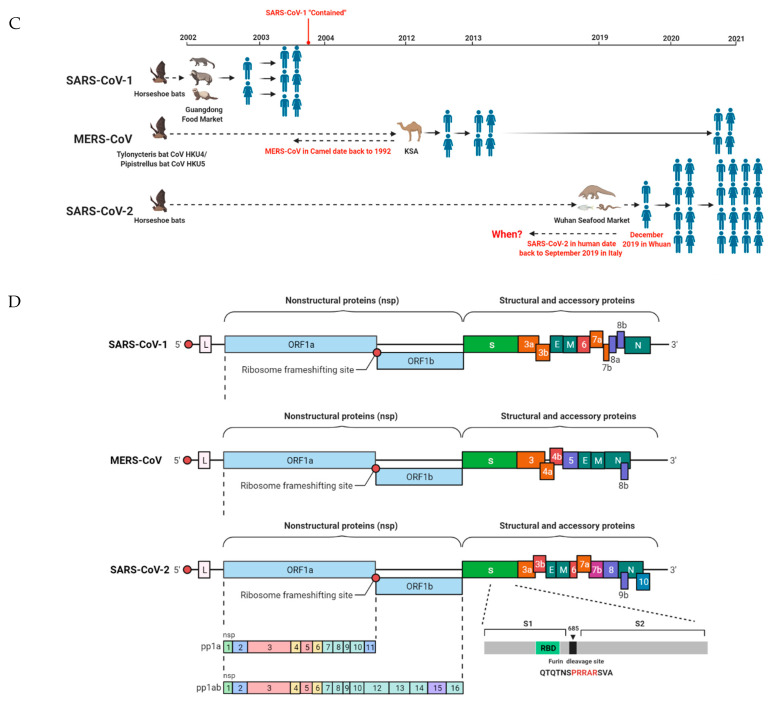
Coronavirus particle, genomes, related family tree and identities of non-structural proteins. The structure of the coronavirus virion (**A**), the coronavirus family tree (**B**), genomes of SARS-CoV-1, MERS-CoV and SARS-CoV-2 (**C**) and timelines for emergence of SARS-CoV-1, MERS-CoV and SARS-CoV-2 (**D**) (created with BioRender.com).

Until the 2002 SARS-CoV-1 outbreak, CoVs were historically thought to only cause mild infections in humans [38]. Before the 2019 SARS-CoV-2 outbreak, SARS-CoV-1 was simply called SARS-CoV. Since the identification of MERS-CoV, the name has been modified several times. In the beginning, MERS-CoV was coined ‘Human Coronavirus Erasmus Medical Center/2012′ (HCoV-EMC/2012) and Novel Coronavirus (nCoV) [39] and because of its similarity with SARS-CoV-1, it was named SARS-like Coronavirus [40]. In order to facilitate the sharing of scientific material, the International Virus Classification Committee’s Coronavirus Research Group named the new coronavirus “Middle East Respiratory Syndrome-Coronavirus” (MERS-CoV) in May of 2013 [41]. SARS-CoV-2 had previously been named 2019-nCoV, while the disease it caused was classified as COVID-19 [42]. Morphologically speaking, CoVs are oval and the capsid of the virus contains Spike (S) glycoproteins, which forms a visual crown effect, hence their “corona” name. The viral particles are between 60 to 140 nm in length and they are single strand, positive sense, RNA viruses (+ssRNA) (Figure 2A).

SARS-CoV-2 has recently become the seventh discovered human CoV [43]. Betacoronaviruses are divided into four lineages including A, B, C and D. SARS-CoV-1 and SARS-CoV-2 are members of the B lineage [12], while MERS-CoV is classified as a member of the C lineage of betacoronaviruses [13] (Figure 2B). The typical CoV genome is 26–32 kb in length and contains at least 9 open reading frames (ORFs) [44]. These ORFs encode a large replicase polyprotein (ORF1a/ORF1b), the surface S glycoprotein, a small envelope protein (E), an outer membrane protein (M), a nucleocapsid protein (N) and several non-structural proteins (nsp). SARS-CoV-1 encodes ORF3a, 3b, 6, 7a, 7b, 8a, 8b and 9b [44]. MERS-CoV expresses ORF3, 4a, 4b, 5 and 8b and SARS-CoV-2 contains ORF3a, 3b, 6, 7a, 7b, 8, 9b and 10 [45,46]. The replicase polyproteins encoded by ORF1a/ORF1b contain two polyproteins PP1a and PP1ab, which are cleaved by a protease called papain-like protease (PLpro), which is encoded within nsp3 [47] and 3C-like protease (3CLpro), which is encoded within nsp5, into 16 nsps, of which the majority have been demonstrated to have specific biological functions. nsp1 of SARS-CoV-1, SARS-CoV-2 and MERS-CoV promotes host mRNA degradation and thus blocks translation [48,49], while the co-expression of nsp3 and nsp4 is required for the formation of double-membrane vesicles (DMVs), the induction of which is essential for viral RNA synthesis [50]. SARS-CoV-1 nsp6 limits autophagosome expansion, which likely promotes viral replication through compromising the ability of autophagosomes to deliver viral components to lysosomes for degradation [51]. Moreover, the SARS-CoV-1 hexadecameric nsp(7 + 8) complex possesses RNA polymerase activity [52]. nsp12 has been identified as another important RNA-dependent RNA polymerase [53]. Similarly, nsp9 of SARS-CoV-1 is an essential protein with RNA/DNA-binding activity and its dimerization is necessary for viral replication [54]. SARS-CoV-1 nsp10 is known to interact with both nsp14 and nsp16, inducing their respective 3′-5′ exoribonuclease and 2′-*O*-methyltransferase activities [55]. CoV helicase nsp13, together with RNA polymerase nsp12 are involved in directing the synthesis of new viral RNA and packaging of new virions consisting of other structural proteins N, S, M and E [56]. Of note, nsp13 possesses extra NTPase activity, which hydrolyzes NTPs and unwinds RNA helices in an NTP-dependent manner [57]. Additionally, SARS-CoV-1 nsp15 is an endoribonuclease with specificity for cleavage at uridylate residues [58]. (Figure 2C). 

## 6. Epidemiology and Transmission

### 6.1. SARS-CoV-1

The first cluster of SARS-CoV-1 cases in 2002 were all linked to a food market in Guangdong, China. Following an investigation of animals at the food market, four of six Himalayan palm civets tested positive for SARS-CoV-1 and antibodies for SARS-CoV-1 were also found in sera of Himalayan palm civets, a raccoon dog and a Chinese ferret badger [59]. Further sequencing of the virus isolated from these animals shared 99.8% similarity to SARS-CoV-1 in humans [59], suggesting zoonotic transmission of the virus at the live animal market. The virus was also thought to have been transmitted from palm civets to humans in a Guangzhou restaurant in late 2003. The restaurant housed palm civets, which all tested positive for the virus. The sequence of the virus isolated from the infected patients was more closely related to that of SARS-CoV-1 in palm civets, than circulating human strains at the time, providing strong evidence for zoonotic transfer of the virus [18]. However, even though SARS-CoV-1 was prevalent among palm civets at animal trading markets, it was not detected in 1107 palm civets subsequently analysed from additional Chinese farms and 21 palm civets captured in the wild [60]. Surprisingly, a CoV similar to SARS-CoV-1 was detected in wild bats in Hong Kong [61]. Further identification of genetically diverse strains of SARS-related CoVs (SARSr-CoVs), isolated from horseshoe bats in different regions of China, provided extra evidence that these mammals are the natural reservoir for SARS-CoV-1 [14]. The current consensus is that palm civets and other animals likely served as intermediate hosts, leading to SARS-CoV-1 infection in humans [62].

After this zoonotic jump, SARS-CoV-1 spread rapidly via human-human transmission. In several outbreaks, “super spreaders” were identified, such as a 26-year-old index patient, who infected 138 others at the Prince of Wales Hospital, Hong Kong [63]. Another outbreak occurred when virus-contaminated faeces, in an apartment-block sewage system, became aerosolised in resident’s bathrooms, infecting 341 people [64]. One hypothesis for this high infectivity of these “super spreaders” is co-infection with other respiratory viruses [65]. Although SARS-CoV-1 was spread mainly via air droplets and physical contact, airborne and fomite transmission also aided its dispersion. SARS-CoV-1 was detected in the air and on surfaces in a patient-occupied room [66]. Similarly, virus-containing aerosols were detected in a ward at the site of a nosocomial outbreak of the virus [67]. The mean incubation period for SARS-CoV-1 was estimated to be 4 days, with most people becoming symptomatic 2–10 days after infection [68]. Asymptomatic SARS-CoV-1 positive cases were also reported [69], with the disease being very mild in teenagers [70]; but increased age and comorbidities, such as diabetes, were associated with more severe illness and a higher risk of mortality [71].

### 6.2. MERS-CoV

MERS-CoV is believed to have also originated in bats [72] and is most phylogenetically related to the Tylonycteris bat CoV HKU4 (Ty-BatCoV HKU4) and Pipistrellus bat CoV HKU5 (Pi-BatCoV HKU5) [15]. Indeed, a 190 nucleotide segment of the MERS-CoV RNARdRp gene, detected in bat faeces in the KSA, was identical to the MERS-CoV sequence isolated from patients in the region [72]. Genetic sequences examined in 2012 and 2013, showed that multiple variants circulated among people and camels. These results indicated that the virus may have spread from multiple animal sources to humans, followed by human to human transmission [19,73]. Several animal sources that live in close proximity to humans were analysed, including camels and goats [74]. Specific anti-MERS-CoV-S protein antibodies were detected in serum samples from camels in Oman and the Canary Islands [75]. Similarly, in the KSA, most serum isolated from camels were positive for S antibodies, but sheep, goats, cattle and chickens were negative [74]. However, pre-existing antibodies did not protect camels from infection [76]. MERS-CoV was mainly found in the respiratory tract and partly in the lower digestive tract of camels. MERS-CoV detected in camel nasal swabs and faeces shares 99.9% sequence similarity to MERS-CoV from humans, with only six nucleotide mutations found in the receptor binding domain (RBD) of the S gene, which did not affect viral binding to cell surface receptors [77]. An analysis of camel blood samples, collected between 1992 and 2010, revealed the existence of MERS-CoV in camels dated back to 1992, despite no reported signs of infection [78]. Since MERS-CoV was found in milk from camels and 9% of camel farm labourers expressed anti-MERS-CoV antibodies, it was hypothesised that the virus was transmitted to humans working in close proximity to camels or regularly consuming camel milk or meat [79]. It was also demonstrated that MERS-CoV can survive in non-sterilised camel milk for 72 h at 4 °C and 48 h at 22 °C; but after heating for 30 min at 63 °C, no active virus was detected [80]. Indeed, a study in 2013 reported a 44-year-old Saudi camel farmer fell ill shortly after treating one of his camels, which had presented with chronic nosebleeds. Genetic analysis of samples taken from the patient and camel in question revealed that their genomes were identical [19]. The latency period of MERS-CoV is usually approximately 5 days. However, it can extend for up to 14 days [81]. Patients with immune dysfunction or other underlying conditions such as diabetes, chronic pneumonia or kidney disease, are more susceptible to MERS-CoV infection and develop critical illness [82]. Healthy individuals only show mild respiratory disease or remain asymptomatic after infection [83].

### 6.3. SARS-CoV-2

Many of the early cases of SARS-CoV-2 in Wuhan suggested that animals sold at the market were intermediate hosts for this novel CoV [84]. However, phylogenetic studies later suggested that SARS-CoV-2 emerged before this time [85,86]. As with other CoVs, bats are thought to be the natural reservoir from which SARS-CoV-2 emerged. Indeed, the bat CoV, BatCoV RaTG13, which shares 96.2% similarity to human SARS-CoV-2, was isolated from horseshoe bats (*Rhinolophus affinis*), as far back as 2013 [16]. Subsequently, two sublineages, related to SARS-CoV-2, were isolated from the Malayan pangolin [87]. The pangolin CoV shares 85.5% to 92.4% sequence similarity with SARS-CoV-2, which is less homology than BatCoV RaTG13. However, the RBD of the pangolin CoV shares 97.4% sequence homology with SARS-CoV-2 and identical amino acids at five critical residues, compared with 89.2% similarity with RaTG13 and only one conserved amino acid, suggesting that pangolins were the source of SARS-CoV-2 zoonotic transmission [87]. Other studies have suggested that even though the pangolin CoV is genetically related to SARS-CoV-2, the pangolin was not the intermediate host responsible for the current pandemic [88,89]; that said, the presence of different strains of CoV in pangolins suggest that they, like bats, could certainly be a natural reservoir for SARS-CoV-2 [17].

The first case of COVID-19 outside of China was recorded in Thailand on 13 January 2020 [90]. However, there was evidence that SARS-CoV-2 emerged and spread from September 2019 in Italy, several months before the first patient was identified in China [91]. Other studies also report that SARS-CoV-2 was detected in human sewage on 27th November 2019 in Brazil, which is earlier than the first reported case in China and there is evidence from serologic testing of blood that SARS-CoV-2 may have been introduced into the United States prior to 19 January 2020 [92,93]. Nevertheless, the virus has spread rapidly across the globe, causing over 100 million cases to date. High viral loads have been detected in the nasal cavity and throat of SARS-CoV-2-infected patients and a viral RNA shedding pattern similar to influenza [94]. Furthermore, the viral loads detected in the upper respiratory tract of asymptomatic patients were comparable to symptomatic patients [94]. Subsequently, a study revealed that infectivity rates were similar between asymptomatic and symptomatic index cases and middle-aged males are more likely to become the ‘silent spreaders’ to transmit the virus [95]. Similarly to SARS-CoV-1, SARS-CoV-2 can remain viable in the air for 1.1 to 1.2 h and on stainless steel and plastic surfaces for 5.6 h and 6.8 h, respectively [96]. This study also showed that SARS-CoV-2 may be viable for a longer period of time on cardboard, compared with SARS-CoV-1 [96]. Characterization of cases from the initial outbreak in Wuhan reported a mean incubation period of 5.2 days [97], which is longer than that of SARS-CoV-1 or MERS-CoV. According to the Chinese Centre for Disease control (CDC), SARS-CoV-2 caused mild to moderate disease in 80.9% of confirmed cases [98]; but risk factors (including comorbidities, such as hypertension, diabetes [36] and age [37]), affected the severity of COVID-19 and increased the risk of mortality.

## 7. Viral Cell Entry and Life Cycle

CoVs can infect a variety of cell types. The heavily glycosylated S protein facilitates cell entry and is the main target for neutralising antibodies. The S protein has a single trans-membrane domain and forms homo-trimeric structures protruding from the virion surface. It contains two functional subunits, including an *N*-terminal outward-facing S1 subunit, which is responsible for receptor binding and the membrane anchored C-terminal S2 subunit, which mediates membrane fusion. The RBD within the S1 subunit displays high genetic diversity among CoVs, while the S2 subunit is highly conserved [99]. Recombinant S protein can induce neutralising antibodies and systemic humoral immune responses in vaccinated mice [100]. Both SARS-CoV-1 and SARS-CoV-2 bind directly to angiotensin converting enzyme 2 (ACE2), enabling cell entry and recruitment of the cellular serine protease, TMPRSS2, for S protein priming [101]. SARS-CoV-2 S protein has an additional furin cleavage site (PRRAR) between the S1 and S2 subunits, which is not present in SARS-CoV-1. The furin-cleaved substrates were also found binding to neuropilin-1 (NRP1), which significantly facilitate SARS-CoV-2 entry and infectivity [102]. SARS-CoV-1 and SARS-CoV-2 S proteins bind with similarly high affinity to ACE2 [101]. During the COVID-19 pandemic, the SARS-CoV-2 S protein has mutated frequently. There are distinct mutation positions, of which D614G is the most prevalent; this single amino acid change lead to higher viral load in the upper respiratory tract of patients but did not affect disease severity [103]. Others mutations include N439K, Y453F, S477N and N501Y in RBD, which increased binding specificity to ACE2, and E484K which facilitated viral “escape” from neutralising antibodies [104,105,106]. Indeed, these mutations have highlighted the importance of the S protein in both viral pathogenesis and vaccine effectivity. ACE2 localises extensively in epithelial cells of alveoli, trachea, bronchi and bronchial serous glands [107]. However, the lack of or lower ACE2 expression in immune cells, colonic epithelial cells and neuronal cells of the brain, contrasts with the confirmed SARS-CoV-1 and -2 infection of these cells. These contradictions suggest that other receptors may be required for infection [108]. SARS-CoV-1 and -2 S protein was reported to bind to the C-type Lectin CD209L, which is expressed on type II alveolar cells and lung endothelial cells, to mediate cell entry [57,109]. Human recombinant soluble ACE2 has been suggested as a potential therapeutic to combat COVID-19, which has already undergone early phase clinical trials for the treatment of ARDS [110]. Remarkably, a main endosomal phosphatidylinositol-3-phosphate/phosphatidylinositol 5-kinase, PIKfyve, which belongs to class III lipid kinase and is involved in endolysosomal system, was reported to participate in virus entry and inhibition of it prevented infection [111]. Furthermore, the basigin (BSG) receptor (also known as CD147), has been identified as an entry receptor for SARS-CoV-2 [112]. Vascular/cardiovascular inflammation and thrombosis occur in severe COVID-19, which may be linked to abundant BSG expression in cardiovascular and renal tissues. Endothelial cell expression of BSG increases with age, which may partially explain the heightened risk of severe disease with age [113]. Notably, BSG is upregulated in a range of diseases that are considered risk factors of severe COVID-19, such as diabetes, obesity, pulmonary hypertension and thrombosis. Gender is also another factor, as males generally has higher BSG expression [114]. More recently, a genome-wide CRISPR screen identified a series of novel host factors involved in phosphatidylinositol phosphate biosynthesis and cholesterol homeostasis, which are crucially required for SARS-CoV-2 infection, including Cathepsin L, transmembrane protein 41B (TMEM41B), transmembrane protein 106B (TMEM106B), cholesterol regulators, ATPases, Retromer, Commander, and Arp2/3 complex [115,116,117,118,119]. These discoveries reveal a complex network associated with viral entry and infection, but highlight several new targets for therapeutic intervention.

Due to the clinical disease similarities during MERS-CoV and SARS-CoV-1 infection, it was thought that MERS-CoV may also utilise ACE2 for cell entry. However, further studies revealed that dipeptidyl peptidase-4 (DPP4, also known as CD26), is bound by MERS-CoV S protein [120,121]. Most respiratory virus infections show a significant ciliated cell tropism. Ciliated cells are widely distributed in the upper and lower respiratory tract, while DPP4 is highly conserved and mainly expressed in the kidney, small intestine, liver, prostate epithelial cells and immune cells (including T cells, activated B, activated natural killer (NK) and myeloid cells) [27]. By comparing the S structures of MERS-CoV and SARS-CoV-(1/2), it was found that both viral core subdomains have high structural similarities, yet still differ in the RBD [122].

**Figure 3:**
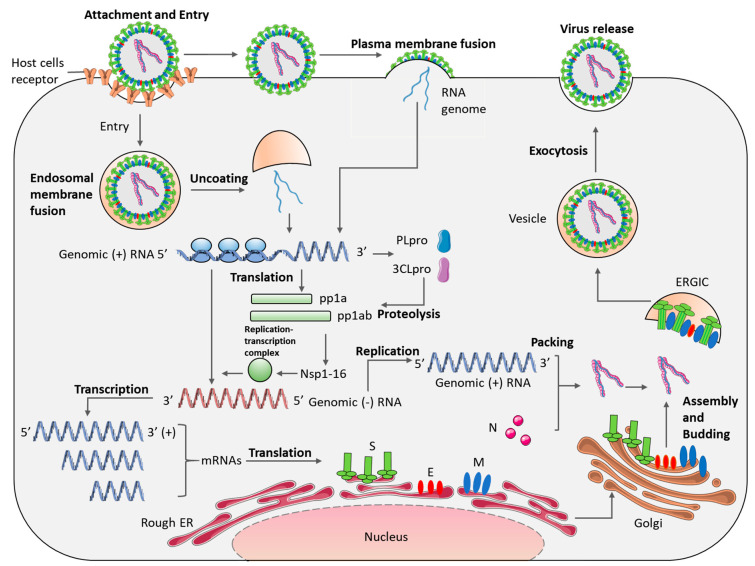
Coronavirus lifecycle. After binding to cellular receptors, coronaviruses enter cells and release their RNA genome. The RNA is transcribed and translated into proteins or replicated to form new RNA, which is assembled into viral particles and released from the cell (ERGIC: ER–Golgi intermediate compartment, ER: endoplasmic reticulum, S: Spike, E: envelope, M: membrane, N: nucleocapsid, nsp: non-structural proteins).

As for the viral life cycle, following receptor binding, the virus is endocytosed. The S protein is cleaved to separate the RBD and fusion domains, facilitating viral envelope fusion with the endosomal membrane and allowing for the RNA genome to be released into the cytoplasm. Some CoVs, such as MERS-CoV, can also enter the cell by plasma membrane fusion [15]. Firstly, viral replicase polyproteins, pp1a and pp1ab, are translated from ORF1a and ORF1b and the synthesis of pp1ab involves programmed ribosomal frame shifting during translation of ORF1a [123]. Papain-like protease (PLpro) and 3C-like protease (3CLpro) cleave pp1a and pp1ab into 16 nsps, that form the viral replicase-transcriptase complex (RTC), which facilitates replication and transcription [123]. The synthesis of full-length negative-strand RNA is initiated by the RTC. During replication, the full-length negative-strand RNA is used as a template to generate the full-length positive-strand genomic RNA. While in transcription, a series of subgenomic mRNAs, which are translated into viral structural proteins, are produced. Once replication and translation are accomplished, the N proteins encapsidate the RNA genome in the cytoplasm, forming the viral nucleocapsids. Meanwhile, the M, S and E proteins are translated in the Endoplasmic Reticulum (ER) and are transported via the Golgi, where budding and particle formation occurs via the ER-Golgi intermediate compartment (ERGIC) [124]. The M protein allows for binding to the nucleocapsids. Ultimately, the particles are transported to the cell membrane and released from the cell by exocytosis in secretory vesicles (Figure 3).

## 8. Innate Immune Response to Coronaviruses

The innate immune system encodes unique pattern recognition receptors (PRRs) for the early detection of viruses and other pathogens. PRRs are expressed mainly by innate immune cells on the cell and endosomal membranes or in the cytoplasm, and recognise pathogen-associated molecular patterns (PAMPs) [125]. Cell surface Toll-like receptors (TLRs) recognise extracellular pathogens, while endosomal TLRs detect pathogens following endocytosis. Intracellular PRRs, including nucleotide oligomerisation domain-like receptors (NLRs), retinoic acid-inducible gene (RIG)-1-like receptors (RLRs) (including RIG-1, melanoma differentiation-associated 5 [MDA5] and laboratory of genetics and physiology 2 [LGP2]) and AIM2-like receptors (ALRs), all detect the presence of intracellular pathogens [126,127]. These PRRs act as early detectors of invading pathogens and their activation initiates intracellular signal transduction pathways, which culminate in the upregulation of pro-inflammatory and anti-viral genes. Anti-viral type I Interferons (IFNs), along with pro-inflammatory cytokines and chemokines are then secreted from the infected cell and mediate the immune response against the invading pathogen.

## 9. TLR and RLR Signalling

Viral nucleic acids are detected by endosomal TLRs, including TLR3, which detects viral double-stranded (ds) RNA, while TLR7 and TLR8 recognise single-stranded (ss) RNA. Following PAMP recognition, TLRs dimerise and recruit adapter proteins, such as myeloid differentiation primary response 88 (MyD88), which leads to downstream activation of TNF Receptor Associated Factor 6 (TRAF6) and subsequent activation of the Inhibitor-κB Kinase (IKK) complex. The IKK complex is made up of IKKα, IKKβ and the regulatory subunit, IKKγ. The IKK complex phosphorylates IκB proteins, marking them a target for ubiquitination and degradation, allowing for the release of the transcription factor, NF-κB. NF-κB translocates to the nucleus and upregulates a plethora of genes, including proinflammatory cytokines and chemokines. TLR7 and TLR8 both signal via the ‘MyD88-dependent pathway’. However, TLR3 utilises another adaptor protein called TIR-domain-containing adapter-inducing interferon-β (TRIF), leading to the recruitment of TRAF3 and subsequent activation of TANK-binding protein (TBK1) and IKK-ε, which leads to subsequent phosphorylation and activation of the interferon regulatory factor (IRF) family of transcription factors, IRF3 and IRF7, thereby upregulating the expression of anti-viral Type I IFNs and interferon-stimulated genes (ISGs) [128,129]. TLR3, which also activates NF-κB via the MyD88-independent pathway, is mainly expressed in hematopoietic cells, particularly in subsets of dendritic cells (DCs), but also in some stromal cells, including airway epithelial cells, and it specifically recognises dsRNA [130].

**Figure 4:**
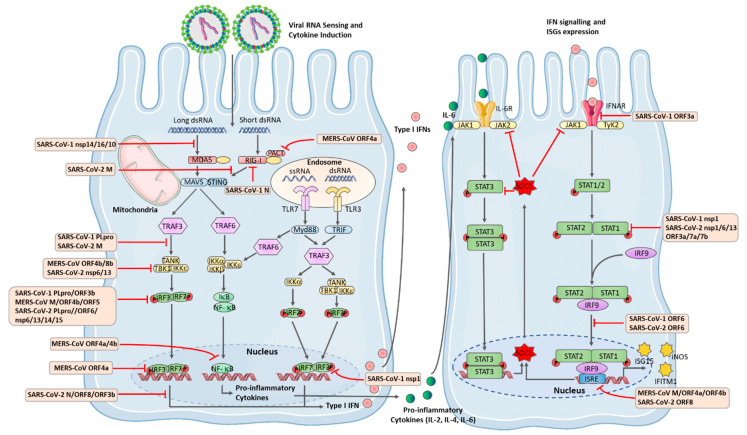
The RLR, TLR and JAK-STAT signalling pathways and interference of signalling pathways by structural and non-structural proteins of coronaviruses. Endosomal TLR3 and TLR7 recognise dsRNA and ssRNA, respectively, leading to activation of NF-κB, which can promote the expression of pro-inflammatory cytokines. TLR3 also triggers IRF-3 activation leading to IFN production. In the JAK-STAT pathway, binding of the receptor leads to its autophosphorylation. Then, Tyk2 and JAK1, which are bound to the intracellular domain of IFNAR, are activated and cause subsequent phosphorylation of STAT1 and STAT2. Phosphorylated STAT1 and STAT2 dimerise and associate with a transcriptional regulator IFR9 (IFN regulatory factor 9), forming the heterotrimeric transcriptional factor complex IFN-stimulated gene factor 3 (ISGF3). Then, ISGF3 translocates to the nucleus and binds to the ISRE promoter of target ISGs. Several coronavirus proteins, which evade anti-viral immune response, are also shown.

Studies suggested that TLR3 provides some protection against MERS-CoV, as mice stimulated with Poly(I:C), a synthetic viral dsRNA, displayed reduced susceptibility to subsequent infection with MERS-CoV, via the upregulation of Type I IFNs [131,132]. Mice lacking the TLR3/TLR4 adaptor protein TRIF, are highly susceptible to SARS-CoV-1 infection and mortality. These mice display dysregulated inflammatory and anti-viral responses to the virus, highlighting the importance of TLR3 in mounting an immune response to SARS-CoV-1 [133]. TLR4 is expressed on the cell surface and recruits MyD88, leading to activation of NF-κB, while following internalization, it initiates TRIF-dependent signalling and downstream IRF activation. TRIF-related adaptor molecule (TRAM), acts as a bridging adaptor for TLR4 and TRIF. As with TLR3, mice lacking TLR4 or TRAM, are also more susceptible to SARS-CoV-1 infection [133]. MyD88 is also required to protect mice against lethality when infected with a murine-adapted SARS-CoV-1 [134]. Additionally, the S glycoprotein of MERS-CoV altered macrophage responses, rendering them hypo-responsive to TLR4 stimulation [135]. Together these findings reveal TLR4 to have an important immune role during CoV infection. TLR7 of plasmacytoid DCs (pDCs), detects the presence of SARS-CoV-1 and MERS-CoV in mice, triggering the induction of Type I IFNs, highlighting an important role for pDCs in the immune response to CoVs [136,137]. RLRs, including RIG-1, MDA5 and LGP2, play a role in the recognition of RNA viruses in the cytoplasm [138,139]. RLRs signal via mitochondrial anti-viral signalling protein (MAVS), which can promote both NF-κB and IRF transcription factor activation (Figure 4). MDA5 has been shown to detect the presence of non-self mRNA during Mouse Hepatitis Virus (MHV), a betacoronavirus which can infect mice, leading to Type I IFN production [140,141]. Recent studies have revealed that MDA5 and LGP2 are the predominant receptors involved in innate immune sensing of SARS-CoV-2 infection in lung epithelial cells [142]; but the produced IFNs were unable to control viral replication in lung cells, which could indicate that other pathways are blocked by the virus [143]. So far, several CoVs proteins have been demonstrated to subvert RLRs signalling through various mechanisms, which are detailed later in this review.

## 10. Anti-Viral IFNs and the JAK-STAT Pathway

Detection of viruses by PRRs triggers the release of IFNs, which promotes viral clearance by inducing apoptosis and inhibiting viral replication [144]. There are three types of IFNs. Type I IFNs are a large subgroup consisting of 13 IFN-α subtypes, IFN-β, ε, κ, τ, δ, ζ, ω, and v [145,146]. The Type II IFN family comprises of IFN-γ [147], while Type III IFNs are comprised of four members, IFN-λ1, IFN-λ2, IFN-λ3 and IFN-λ4 [148]. IFNs signal via binding to specific receptors. Type I IFNs bind IFN-α receptors (IFNARs) [149], Type II IFN binds to the IFN-γ receptors (IFNGRs) [147], and Type III IFN signals by a heterodimeric receptor complex containing IFNLR1 and IL10RB [150]. TLR and RLR signalling leads to the activation of the transcription factors, IRF3, NF-κB and AP1, which cooperatively bind to the promoter region of IFN-β and upregulate its gene expression [151]. IFN-β is released by cells and binds to IFNARs on target cells, inducing the expression of IFN-α, in a positive feedback loop. Following receptor recognition, IFN-α/β signal via the Janus kinase/signal transducers and activators of transcription (JAK/STAT) pathway (Figure 4). Ligand binding induces receptor autophosphorylation followed by phosphorylation and activation of receptor bound JAK1 and tyrosine kinase 2 (Tyk2). STAT proteins are then recruited to the receptor, leading to their phosphorylation on specific tyrosine residues. Phosphorylated STAT proteins form homo- or heterodimers which translocate to the nucleus and upregulate the expression of target ISGs. STAT1 and STAT2 form a complex with IRF9, called the IFN stimulated gene factor 3 (ISGF3), which binds to the IFN stimulated response element (ISRE) of target genes. STAT3 is also required for the upregulation of a subset of ISGs [152]. Type I IFNs induce the expression of over 300 ISGs, with anti-viral properties that limit viral replication [153]. One such ISG is ISG15, which catalyses the conjugation of ISG onto target proteins and inhibits viral replication [154]. Inducible Nitric Oxide Synthase (iNOS), stimulates the production of NO, which has been shown to inhibit SARS-CoV-1 replication [155]. Interferon-inducible transmembrane proteins 1, 2, and 3 (IFITM1, 2, and 3), have been shown to restrict SARS-CoV-1 replication and cell entry in vitro [156] and Lymphocyte Antigen 6 Family Member E (LY6E), has recently been shown to inhibit SARS-CoV-1&2 and MERS-CoV cell entry [157]. Intriguingly, ACE2 is also an ISG, that is significantly upregulated by Type I IFN. However, IFN-induced ACE2 is not thought to enhance SARS-CoV-2 replication in the presence of IFN-induced anti-viral activity, in primary human bronchial epithelial cells [158]. JAK/STAT signalling is also employed by other cytokines such as IL-6; indeed IL-6 is commonly elevated in COVID-19 patients and tightly correlated with disease severity [34]. Of additional importance, a SARS-CoV-2 hamster model presented STAT2-drived exuberant immune responses and progressed to severe lung injury during infection [159]. In summary, despite its remarkable anti-viral effects, in certain circumstances, the JAK/STAT pathway can be detrimental, and its dual roles should be considered during clinical management.

Negative feedback of the JAK/STAT pathway appropriately tunes its responses. In recent years, several groups have explored negative regulators induced by type 1 IFN signalling, including suppressor of cytokine signalling (SOCS), protein inhibitors of activated STAT (PIAS), protein tyrosine phosphatase (PTP) and ubiquitin-specific peptidase 18 (USP18). SOCS1 has been revealed as a potent inhibitory modulator, whose expression results in decreased phosphorylation of Tyk2, JAK1, STAT1 [160]. In fact, a broad range of viruses utilise both SOCS1 and SOCS3 to block JAK/STAT signalling; for example, coronavirus transmissible gastroenteritis virus (TGEV) dampened the IFN-I anti-viral response and facilitated TGEV replication through increased SOCS1 and SOCS3 expression [161]. Intriguingly, SARS-CoV-1 S protein has been demonstrated to induce SOCS3 [162]; and, although there is no direct evidence that SARS-CoV-2 induces SOCS1/3, it has been suggested that their expression might be used as an effective prophylactic and/or therapeutic against COVID-19 [163].

## 11. SARS-CoV-1 Immune Modulatory Mechanisms

CoVs have evolved strategies to evade host immune responses and promote viral replication. SARS-CoV-1 induces the formation of complex DMVs of modified ER in which RNA replication takes place, which may prevent nucleic acid sensors from detecting the presence of viral RNA [164]. SARS-CoV-1 avoids MDA5 detection through mediating self-viral mRNA cap methylation under the collaboration of nsp14, nsp16 and nsp10 [165], preventing MDA5 from recognising viral mRNA as foreign [141]. SARS-CoV-1 has also been reported to inhibit the activation of IRF3, thus attenuating IFN-β production [166]. Indeed, SARS-CoV-1 N protein, ORF3b and ORF6 all inhibited the induction of IFN-β [167]. N protein competes with RIG-I for binding to tripartite motif protein 25 (TRIM25), thereby preventing the ubiquitination and activation of RIG-I [168]. SARS-CoV-1 nsp1 attenuates type I IFN production by targeting type I IFN mRNA for degradation [169,170]. The SARS-1-CoV PLpro is a domain within nsp3, which functions as a deubiquitinase and can remove ISGylation from target proteins [171,172]. Protein conjugation by the ubiquitin-like ISG15 is termed ISGylation. The RIG-I adaptor protein, MAVS, signals via a stimulator of interferon genes (STING)—TRAF3—TBK1:IRF3 complex to induce IFN-β [173,174,175,176]. The immune-suppressive nature of PLpro can be seen in its broad reduction in RIG-I, STING, TRAF3, TBK1 and IRF3 ubiquitination, through its deubiquitination activity [177]. SARS-CoV-1 PLpro can subvert IRF3-mediated IFN induction, after IRF3 phosphorylation and nuclear translocation, by a mechanism that requires its deubiquitinating properties [178]. Indeed, SARS-CoV-1 PLpro also interacts with this complex, in which the association of PLpro with STING reduces the formation of STING dimers, preventing its ubiquitination and interaction with MAVS and TBK1 and subsequent induction of IFN-β [179]. A study also revealed that the catalytic ability of PLpro is indispensable for inhibiting IFN-β production [47]. Indeed, the various functions of PLpro indicate that it is a prominent IFN antagonistic.

SARS-CoV-1 is capable of inhibiting type I IFN signal transduction. ORF3 was found to induce serine phosphorylation of IFNAR1 and promote ubiquitination and degradation [180]. ORF3b and ORF6 have been shown to inhibit ISRE promoter expression in response to IFN stimulation, revealing that they inhibit type I IFN signalling. Furthermore, ORF6 has been shown to inhibit the nuclear translocation of STAT1 in response to IFN-β [167]. Indeed, ORF6 binds and sequesters the nuclear import protein karyopherin-α2 (KPNA), thus preventing its availability to shuttle STAT1 to the nucleus [181]. SARS-CoV-1 nsp1 interferes specifically with STAT1 phosphorylation, thus attenuating the anti-viral IFN immune response [182]. SARS-CoV-1 infection also leads to tyrosine dephosphorylation of STAT3 in Vero cells [183]. Intriguingly, a recent study demonstrated that STAT3 plays an anti-viral role in type 1 IFN signalling [152], which suggests a specific mechanism used by SARS-CoV-1 to block anti-viral activity.

pDCs are known as type 1 IFN-producing cells and have the ability to secrete massive amounts of type 1 IFN following viral stimulation [184]. However, DCs from patients with SARS-CoV-1 produced low levels of IFN-β, but high levels of the chemokine CXCL10 [185]. Similarly, SARS-CoV-1 has been shown to inhibit the induction of IFNs, RANTES, IL-6 and ISGs, but upregulate CXCL10 and CXCL8 chemokines in intestinal epithelial cells [186,187]. However, it is thought that the upregulation was caused by increased DNA-binding activity of AP-1 and NF-κB [188], which is in contradiction with the downregulated IL-6 and Regulated upon Activation, Normal T cell Expressed and Secreted (RANTES, also known as CCL5). Conversely, SARS-CoV-1 N protein has been reported to activate AP-1, but not NF-κB in hepatocytes and Vero cells [189]. Similarly, SARS-CoV-1 S protein can activate MAPK signalling, leading to AP-1-mediated CXCL8 induction in lung epithelial cells [190]. Indeed, CXCL8 and CXCL10 are markers of SARS-CoV-1 infection and may contribute to disease pathogenesis through recruitment of immune cells to the lungs. In patients with SARS-CoV-1, high levels of CXCL10, CXCL8, IL-6 and monocyte chemoattractant protein-1 (MCP-1) were observed in the blood and lung tissue, along with macrophage and monocyte infiltration and lymphocyte depletion [191]. Elevated levels of CXCL10, CXCL8 and monokine induced by IFN-γ (MIG-γ) were associated with poor clinical outcomes in SARS-CoV-1 patients [192] and were higher in patients where the disease was fatal, compared with those who recovered [193]. Immune cell infiltration, caused by chemokines, may contribute to the disease pathology observed in severe SARS-CoV-1 patients. Indeed, patients with severe COVID-19 were found to have higher levels of chemokines, such as CXCL10 and MCP-1 [34].

## 12. MERS-CoV Immune Evasion

MERS-CoV has the highest associated mortality rate, possibly due to the significant dysregulation of the host transcriptome that it causes, which is much greater than SARS-CoV-1 [194]. An in vitro study also identified that, compared with SARS-CoV-1 and SARS-CoV-2, MERS-CoV led to higher viral replication in the lungs and subsequently increased production of proinflammatory cytokines [195]. This higher level of replication was possibly due to enhanced viral protein virulence. MERS-CoV ORF5, ORF4a, ORF4b and the M proteins are thought to play pivotal roles in IFN suppression, with ORF4a being the most effective. ORF4a inhibits both IFN production (measured via IFN-β promoter activity, IRF-3 function and NF-κB activation), and STAT activation of the ISRE [196]. ORF4 also binds and antagonises the dsRNA-binding protein, PRKRA (interferon-inducible double-stranded RNA-dependent protein kinase activator A, also known as PACT), which acts as a cellular activator of RIG-I and MDA5 to facilitate an innate anti-viral response in the cytosol [197]. Indeed, MERS-CoV without ORF3, 4a, 4b and 5 (dORF3–5) had a diminished replication capacity and robust IFN responses, when compared to the wild-type virus, in human epithelial cells. The dORF3–5 infection also caused higher levels of pro-inflammatory cytokines secretion, than that wild-type virus, revealing the role of these proteins in suppressing the inflammatory response [198]. Additionally, MERS-CoV ORF4b was found to localise in the nucleus and robustly inhibit RIG-I-mediated induction of the IFN-β promoter via IRF3 [199]. ORF4b specifically binds to TBK1 and IKKε, resulting in the inhibition of IRF3 phosphorylation [200]. ORF4b-encoded protein was also found to inhibit the nuclear translocation of p65 through association with KPNA4, which is known to assist in the translocation of the NF-κB complex into the nucleus [201]. ORF4b also contributes to viral replication. Viruses isolated from camels with deletions in ORF4b showed impaired replication and higher type I and III IFN responses in human cells [202], suggesting that ORF4b has a role in antagonising the anti-viral response, thus enabling viral pathogenesis. ORF8b suppresses type I IFN expression, by competing with IKKε for HSP70 interaction, which is required for the activation of IKKε and IRF3 [203]. ORF8b was also found to sequester MDA5-mediated NF-κB activation, RLR-activated IRF3 phosphorylation and CARD-CARD interactions between RIG-I and MAVS [204,205]. MERS-CoV-nsp1 inhibits host gene expression by selectively targeting mRNAs to the nucleus and promoting their degradation [48], while the nsp3–4 polyprotein induces the formation of DMVs, which are associated with viral RNA replication [50]. MERS-CoV PLpro, which is encoded within nsp3, is a multifunctional enzyme with protease, deubiquitinating and deISGylating activities [47]. A mutant of MERS-CoV lacking PLpro was more sensitive to type I IFN in an IFN-induced proteins with tetratricopeptide repeats (IFIT)-dependent manner, providing an attenuation mechanism [206].

During infection, MERS-CoV was found to specifically downregulate the expression of several genes within the antigen presentation pathway, including both type I and II major histocompatibility complex (MHC) genes, revealing its ability to suppress engagement of the adaptive immune response [194]. Subsequent depletion of both CD4+ and CD8+ T cells resulted in suboptimal MERS-CoV clearance, highlighting the importance of these adaptive immune cells in combating this virus [137]. MERS-CoV also downregulates Th1 and Th2 cytokines and chemokines, leading to severe infection, again revealing how MERS-CoV suppresses the essential adaptive immune response [207]. While research has discovered several MERS-CoV protein functions, the complexity of MERS-CoV infection and its interaction with both the innate and adaptive immune systems remains largely unknown.

## 13. SARS-CoV-2 Antagonism of IFN

Type I IFN deficiency in the blood has become a hallmark of severe COVID-19 [208] and SARS-CoV-2-infected primary human bronchial epithelial cells displayed a limited IFN-I and IFN-III response, with only a small subset of ISGs induced [209]. Although SARS-CoV-2 has been reported to be more sensitive than SARS-CoV-1 to type I IFN [210], SARS-CoV-2 infection still triggers delayed immune responses [180]. Several key viral proteins which act as IFN antagonists of SARS-CoV-2 might be an explanation for impaired IFN responses. Notably, SARS-CoV-2 PLpro, has been shown to cleave ISG15 from IRF3, of which the ISGylation is essential to maintain IRF3 activation [211], thus attenuating type I IFN responses [212]. SARS-CoV-2 ORF6 is a more potent IFN antagonist; its *C*-terminus interacts with KPNA2, which regulates nuclear import, thus blocking nuclear translocation of specific transcription factors, indeed, it inhibited both IRF3-mediated IFN induction and STAT1-mediated signal transduction [213]. An interactome study identified ORF6 interaction with a peripheral nucleoporin, Nup98 [214]; ORF6 localises at the nuclear pore complex (NPC) and directly interacts with Nup98 via its *C*-terminal domain, which disrupts STAT1 nuclear import, thus blocking IFN signalling [215]. ORF8 and N proteins also inhibit type I IFN induction, while ORF8 can additionally inhibit IFN-β-mediated ISRE activation [216]. ORF8 also down regulates MHC-I molecules, suggesting a potential role in regulating adaptive immunity [217]. Consistent with the in vitro studies, SARS-CoV-2 variants that lack ORF8 expression are associated with milder symptoms [218]. Furthermore, nsp13, nsp14 and nsp15 proteins of SARS-CoV-2 potently suppress IFN production by inhibiting IFR3 nuclear localisation [219]. Other studies revealed that TBK1 and IRF3 phosphorylation were suppressed upon nsp6 and nsp13 expression [220]. Furthermore, the tyrosine phosphorylation of STAT1 and STAT2 was reduced by nsp1, nsp6, nsp13, ORF3a, ORF7a and ORF7b. Surprisingly, SARS-CoV-2 nsp1 is more efficient than those of SARS-CoV-1 and MERS-CoV at suppressing type I IFN signalling, via its blockade of STAT phosphorylation [220]. ORF3 a can induce apoptosis [221], while a mutation in ORF3a is associated with higher infection and mortality rate [222]. Interestingly, viral and host protein–protein network analysis predicts that the functions of ORF3 associate with JAK/STAT pathway components, including JAK1, JAK3 and STAT3 [222]. Indeed, SARS-CoV-2 ORF3b was demonstrated to have a more potent antagonism of type I IFN activation than SARS-CoV-1 ORF3b, which is likely due to *C*-terminal truncation of the SARS-CoV-2 ORF3b [223]. A recent study also reported that SARS-CoV-2 membrane (M) protein interacted with RIG-I, MAVS, and TBK1, preventing the formation of the multiprotein complex containing RIG-I, MAVS, TRAF3, and TBK1, thus inhibiting the production of type I and III IFNs [224]. Of additional importance, ORF10 is involved in the ubiquitination proteasome pathway, due to its interaction with the Cullin-2 protein; and nsp8 is suggested to hijack the Sec61-mediated protein translocation pathway for entry into the ER [214]. In addition, clinical studies have identified neutralising autoantibodies against IFN-α2 and/or IFN-ω in severe COVID-19 patients, which were not detected in asymptomatic nor healthy individuals, nor in patients with mild infection, possibly revealing an additional immune evasion mechanism that the virus uses to avoid elimination [225]. Another clinical study reported that mild COVID-19 patients display elevated ISG expression across every blood cell population and these cells are absent in patients with severe disease, which maybe due to specific antibodies against ISG-expressing cells [225]. Importantly, SARS-CoV-2 has a lower mortality rate, but is more transmissible and therefore has caused a greater number of fatalities worldwide. Indeed, the robust replication competence of SARS-CoV-2 in human upper respiratory bronchus might explain its efficient transmission among humans [195].

## 14. Treatment

### 14.1. SARS-CoV-1

During the SARS-CoV-1 epidemic, the main cause of death was ARDS, which arose in 16% of patients; patients whose disease progressed to ARDS had a 50% chance of mortality [10]. During the SARS-CoV-1 outbreak, several therapies were used, including ribavirin, corticosteroids, anti-retroviral protease inhibitors and IFN-α. It is notable that the efficacy of these treatment strategies was difficult to determine due to the lack of controlled parameters. The guanosine analogue, ribavirin, was used due to its effectiveness against many RNA and DNA viruses. Clinical evidence to support the use of ribavirin was not found and in some cases led to side effects, such as haemolytic anaemia [226], resulting in its use not being advised [227]. Immunosuppressive corticosteroids were also used, which in some cases led to complications, such as aspergillosis [228,229]. Furthermore, early corticosteroid treatment resulted in enhanced SARS-CoV-1 viral load [230], although in other cases it was seen as beneficial in combination with mechanical ventilation [231]. One study showed a positive response of patients to co-treatment with IFN-α and corticosteroids, compared with corticosteroids alone, including quicker resolution of lung pathology [232]. However, IFN-α treatment was shown to have no effect in another study [231]. Methylprednisolone treatment had favourable clinical outcomes, although the study lacked a comparative control [233]. Treatment with lopinavir/ritonavir anti-retroviral protease inhibitors and ribavirin had good outcomes, with a reduced SARS-CoV-1 viral load and alleviated symptoms [234,235]. Furthermore, the use of convalescent plasma, serum or hyperimmune immunoglobulin appeared to reduce mortality and was deemed safe to use [236].

Vaccines against SARS-CoV-1 infection have been developed by various groups using different techniques, such as whole-virus inactivation, virus-like particles, recombinant viral vectors expressing SARS S protein and DNA-based vaccines [237,238,239,240,241,242,243]. One inactivated virus vaccine and a DNA-based vaccine have been evaluated in phase I clinical trials and were shown to elicit T cell responses and antibody responses against SARS-CoV-1 antigens [244,245].

### 14.2. MERS-CoV

Due to the similarity with SARS-CoV-1, similar anti-viral treatments were implemented on patients with MERS-CoV. There are several therapeutic strategies in development, including virus replication inhibitors, DPP4 inhibitors and immune modulators. To date, patients were often given a combination of treatments, including respiratory therapy and anti-viral drug therapy. To ensure effective treatment, it is recommended that patients should receive treatment immediately after the diagnosis. IFN-α2a, IFN-α2b and IFN-β1a, are considered as effective anti-viral treatments. In one study of MERS-CoV-infected rhesus macaques, the combination of IFN-α2b and ribavirin reduced virus replication, moderated the host response and improved clinical outcomes [246]. Moreover, monotreatment with lopinavir/ritonavir or IFN-β1b, or in combination, improved outcomes of MERS-CoV infection in a non-human primate model of common marmosets [247]. MERS-CoV was found to be 50–100 times more sensitive to IFN-α treatment than SARS-CoV-1 in vitro, an observation that may have important implications for the treatment of patients with MERS-CoV [248]. Another study found that IFN treatment could be protective or pathogenic depending on the time at which the treatment was given, which highlights the importance of timely IFN treatment during infection [137]. Other research has pointed to the potential use of a DPP4 inhibitor to modulate the pathogenesis of MERS-CoV infection [249].

Neutralising antibodies (nAbs) were detected in sera from MERS survivors; these antibodies that was able to affect virus clearance in a murine model; interestingly the sera showed low antibody titres in patients with less severe disease [250]. It is noteworthy that several human nAbs were identified with binding domains in the viral S protein–receptor interface, that block virus attachment [251]. Unfortunately, there remains no licensed vaccine for MERS-CoV infection. Since camels are naturally reinfectable with MERS-CoV, their vaccination has been investigated [252]. A phase I clinical trial of a modified vaccinia ankara (MVA)-based MERS vaccine has shown safety in adults, along with antibody and T cell responses against MERS S in most participants [253].

### 14.3. SARS-CoV-2

Ongoing investigations are identifying treatment strategies for SARS-CoV-2. In vitro investigations indicate that SARS-CoV-2 is more sensitive to treatment with type I IFN [254,255], highlighting its therapeutic potential; trials using type I IFNs are ongoing. Meanwhile, clinical improvement was seen in 68% of a cohort of severe COVID-19 patients treated with compassionate use remdesivir [256]. Remdesivir has been demonstrated to significantly improve time to recovery and reduce disease progression in patients needing oxygen, compared to placedo and was approved by FDA [257]. Chloroquine, a widely used anti-malaria drug with proven anti-viral effects for HIV and SARS-CoV-1, has been shown to inhibit SARS-CoV-2 infection in vitro [258]. Initially, hydroxychloroquine, a less toxic derivative of chloroquine, was thought to have potential as a treatment as it seemed to attenuate SARS-CoV-2 infection, while suppressing harmful inflammatory responses [259]. However, hydroxychloroquine had no beneficial effects during subsequent clinical trials; it has not been approved for treatment [259,260]. Due to the immunopathology of COVID-19, with evidence of a cytokine storm in patients with severe disease, timely treatment with anti-inflammatories has been recommended [261]. Treatment with convalescent plasma has also shown promise in patients with severe COVID-19, which improves clinical symptoms within 3 days [262]. Regeneron’s monoclonal antibodies, casirivimab and imdevimab, have been granted FDA approval for the treatment of patients with mild to moderate COVID-19 [263]. Other potential treatments include soluble ACE2, which has previously shown promise for the treatment of ARDS [110], or the serine protease, Camostat, to inhibit SARS-CoV-2 S protein priming by TMPRSS2 [101,264]. Surprisingly, the anti-parasitic drug, ivermectin, also shows some anti-SARS-CoV-2 effects in vitro [265]. There is also some evidence that the tuberculosis vaccine Bacillus Calmette–Guerin (BCG), an attenuated strain of *Mycobacterium bovis*, provides some protection against COVID-19 [266]. There are several vaccines available for SARS-CoV-2 and efforts continue to develop additional candidates, with several focused on providing protection against new mutant strains. One particular importance is the use of novel mRNA vaccine technology. Pfizer and Moderna have both developed new mRNA vaccines that report to provide unprecedented protection [267]. We have included a summary of promising treatment strategies for therapeutic and prophylactic interventions against SARS-CoV-1, MERS-CoV and SARS-CoV-2 infection in Table 2.

## 15. Conclusions and Future Considerations

The current rapidly evolving pandemic situation represents a huge challenge to scientists and medical professionals. There are still many aspects of CoVs that remain unclear, including zoonotic origins, genetic evolution, interaction between the virus and host and effective treatment strategies. Indeed, SARS-CoV-2 continues to infect globally, with increasing numbers each day, and MERS-CoV remains a threat. With the ever-evolving reservoir of CoVs in bats, future zoonotic events are likely and could result in a novel epidemic or pandemic in the years to come. Indeed, the likelihood of a fourth deadly CoV emerging in our future remains high, highlighting the need for renewed efforts in solving outstanding questions. The continued threat from intermediate animals and changes in climate may both affect the distribution of disease vectors. Understanding how these CoVs transmitted from animals to humans is essential. Similarly, both the SARS-CoV-1 epidemic and SARS-CoV-2 pandemic highlight the danger of trading animals which could harbour infectious diseases. Finally, the currently known mechanisms of immune evasion utilised by CoVs and the complex anti-viral immunity network reviewed in this paper, highlight the continued need to understand the interplay between CoVs and the immune response. In fact, a comprehensive understanding of these viruses and their interplay with the immune response will be a powerful weapon to fight existing and future deadly CoVs.

## Figures and Tables

**Table 1:** Biological and Epidemiological Characteristics of CoVs (10 Mar 2021).

		SARS-CoV-1	MERS-CoV	SARS-CoV-2
Genome type		ssRNA(+)	ssRNA(+)	ssRNA(+)
Genome size		29.7 kb	30.1 kb	29.9 kb
Genus		Beta-CoV, linage B [12]	Beta-CoV, linage C [13]	Beta-CoV, linage B [12]
Origin		Guangdong, China	Saudi Arabia	Wuhan, China
Possible natural reservoir		Horseshoe bats [14]	Tylonycteris and Pipistrellus bats [15]	Horseshoe bats or Pangolin [16,17]
Possible intermediate host		Civet cat [18]	Dromedary camel [19]	Pangolin [17]
Epidemiological Characteristics	Total laboratory-confirmed cases	8089	2566	117,764,619
	Infected countries	29	27	215
	Gender (% of male)	47% [20]	78% [21]	53% [22]
	Median age of patients	43 [20]	55 [21]	61 [22]
	Reported death number	774	881	2,613,747
	Mortality	9.6%	34.4%	0.8–10.8%
Latency period		2–10	5–14	12–20
Clinical Symptoms		Acute respiratory distress syndrome (ARDS)	Acute respiratory distress syndrome (ARDS)	Fever, cough, loss of taste, shortness of breath, sore throat and dyspnoea
Functional receptor		Human angiotensin-converting enzyme 2 (ACE2), CD209 L	Human dipeptidyl peptidase 4 (DPP4 or CD26)	Human angiotensin-converting enzyme 2 (ACE2), CD209 L, NRP1, PIKfyve, BSG
Receptor localised organ		Lungs, intestines, kidneys, heart, brain liver and testicles [23]	Brain, heart, lung, kidney, spleen, intestine, and liver [24]	Lungs, intestines, kidneys, heart, brain liver and testicles [23]
Receptor distributed cell lines		Lung alveolar epithelial cells, enterocytes of the small intestine, arterial and venous endothelial cells and arterial smooth muscle cells [25]	Bronchiolar epithelium cells, alveolar interstitium cells, kidney vascular smooth muscle cells and immune cells [26,27]	Lung alveolar epithelial cells, enterocytes of the small intestine, arterial and venous endothelial cells and arterial smooth muscle cells [25]

**Table 2:** A summary of coronavirus therapeutic research.

Strategy	Treatment	Mechanism	Efficacy			Side Effect/Disadvantage
			SARS-CoV-1	MERS-CoV	SARS-CoV-2	
**Serine protease inhibitor**	Camostat mesylate	Block the entry of virus into TMPRSS2-expressing cells	Significantly reduced the entry of pseudotyped SARS-1-S (1–5 μM) [101]	Significantly reduced the entry of pseudotyped MERS-S (1–5 μM) [101]	Significantly reduced the entry of pseudotyped SARS-2-S and authentic infection (1–5 μM) [101]	The drug has few and mild side effects even at high dosages for other diseases [268]. Currently in many phase I and phase 2 clinical trials
**Nucleoside inhibitor**	Ribavirin	Stops viral RNA synthesis and viral mRNA capping [269]	No demonstrable anti-viral activity	Reduced virus replication in susceptible Vero cell lines [270]	Ribavirin was recommended by Chinese National Health Commission because of its in vitro effect [271], but no significant benefit was observed with ribavirin treatment for COVID-19 patients later [272]	Haemolytic anaemia, hypocalcemia and hypomagnesmia in SARS-CoV-1 infection [226] MERS-CoV infected Vero cells displayed a high level of resistance to the activity of ribavirin [270]
**RNA polymerase inhibitor**	Remdesivir	Inserts into viral RNA chains, causing their premature termination	Highly inhibited viral titres [273]	Highly inhibited viral titres [273]	Significantly improved time to recovery and reduced disease progression in patients needing oxygen, compared to placebo, and was approved by FDA [257] for emergency use	Approximately 10% healthy volunteers had raised blood levels of liver enzymes. Another common effect is nausea [274]
	Favipiravir	Inhibits the RNA-dependent RNA polymerase (RdRp) of RNA viruses	Weak inhibitory effects on MERS-CoV RdRp activity [275]	No reports	Significantly improved the latency to cough relief and decreased the duration of pyrexia in moderate COVID-19 patients [276]	Increased serum uric acid and Psychiatric symptom reactions in COVID-19 patients [276] and clinical trials are ongoing
**HIV Protease inhibitors**	Lopinavir/ ritonavir	Inhibits viral replication	Reduced use of pulse methylprednisolone, milder disease course and reduced viral load [234]	Improved clinical, radiological, and pathological outcomes and lower mean viral loads in lung tissues [247]	No benefit was observed with lopinavir–ritonavir treatment [277]	Gastrointestinal adverse events including nausea, vomiting, and diarrhoea were more common in lopinavir–ritonavir group [277]
	Nelfinavir	Inhibits viral replication	Strongly inhibited viral replication in Vero E6 cells [278]	No activity found in vitro [279]	Potential inhibitor against main protease (Mpro) [280] Did not reduce viral load in the lungs of SARS-CoV-2-infected hamsters, but markedly improved lung pathology despite a massive infiltration of neutrophils [281]	Limited clinical data
**3 CLpro inhibiotr**	α-ketoamide inhibitors	Inhibit viral replication	Exhibited excellent anti-MERS-CoV activity in virus-infected Vero E6 cells [282]	Exhibited excellent anti-MERS-CoV activity in virus-infected Huh7 cells [282]	Inhibited SARS-CoV-2 replication in human Calu-3 lung cells [283]	No human proteases with a similar cleavage specificity are known; such inhibitors are unlikely to be toxic [283]
**PIKfyve kinase inhibitor**	Apilimod	Inhibit viral replication during entry	Inhibited MERS pseudotyped particles entry and replication in Vero E6 cell [284]	No reports	Strongly blocked SARS-CoV-2 infection in Vero E6 cells [111]	Phase I and phase II clinical trials of other diseases have shown that apilimod is safe and well tolerated [111]
**Anti-viral compound**	Arbidol	Prevents viral entry into the target cells	Reduced virus reproduction in vitro [285]	No reports	Patients had a shorter duration of infection (positive RNA test results), compared to those in the lopinavir/ritonavir group [286]	A clinical trial in patients with COVID-19 has been initiated in China [287]
**Anti-parasitic agent**	Ivermectin	Inhibits viral replication	No reports	No reports	5000-fold reduction in SARS-CoV-2 RNA levels in vitro [265] Patients treated with ivermectin recovered earlier from hyposmia/anosmia [288]	No noticeable side effects [289] and larger clinical trials are needed
**Anti-inflammatory agents**	Corticosteroids	Blocks the action of inflammatory mediators and induce anti-inflammatory mediators [290]	Delayed viral clearance in blood [230]	Delayed viral clearance in respiratory tract [291]	Routine corticosteroids should be avoided unless they are being used to treat another condition (WHO guideline) [292]	Secondary bacterial/fungal infections, hyperglycemia, electrolyte imbalance and psychosis in SARS-CoV-1 infection treatment [230]
	Chloroquine/hydroxychloroquine	Inhibits the production of inflammatory cytokines [293] and interferes with terminal glycosylation of the cellular receptor [294]	Ameliorated the hyperinflammatory response induced by viral infection [293] Prevented the spread of SARS-CoV-1 in cell culture [294]	Effectively blocked viral infection in vitro [295]	Several randomised controlled trials showed potential effects in reducing respiratory symptoms and pulmonary inflammation in COVID-19 patients [296]	High doses of CQ/HCQ in COVID-19 patients can be associated with increased cardiac adverse events [297]
	Tocilizumab	IL-6 receptor antagonist	No reports	No reports	Decreased the mortality rate in severe COVID-19 patients [298]	Some reports showed an increase in hepatic enzymes (29%), thrombocytopenia (14%), and serious bacterial and fungal infections (27%) [299]
	Anakinra	IL-1 receptor antagonist	No reports	No reports	Averted the need for mechanical ventilation in patients with severe COVID-19 pneumonia. Significantly reduced biomarkers of inflammation [300]	A three times increased level of aminotransferase in liver in 13% patients [301]
	Statins	Decreases inflammation and proinflammatory cytokines production	No reports	No reports	Statins can reduce inflammation and the progression of lung injury in experimental models. Statin use was associated with improved survival l [302]	Statins are first-line lipid-lowering therapies, with well-tolerated side effects. Limited clinical data [302]
**Anti-viral cytokine** **(type I interferon)**	Interferon-α	Anti-viral ISGs induction inhibits viral replication and production	IFN alfacon-1 (recombinant synthetic type I IFN) was well tolerated by patients [232] PEG-IFN-α-2 b inhibited symptoms in macaques [303]	Inhibited viral induced cytopathic effect and RNA levels in vitro [248]	Early usage of IFN-α2 b decreased in-hospital death, but late usage raised mortality [304]	Mild neutropenia and some elevation of serum transaminase levels in SARS-CoV-1 infection [232]
	Interferon-β	Anti-viral ISG induction inhibits viral replication and production	IFN-β-1 b inhibited viral replication more effectively than IFN-α-2 b in vitro and showed prophylactic protection and anti-viral ability [305]	Improved clinical, radiological, and pathological outcomes and lower mean viral loads in lung tissues [247]	Early administration significantly reduced mortality in severe COVID-19 patients [306]	Injection-related side effects (fever, chills, myalgia, and headache) a few hours after injection of IFN, happened in 19% of COVID-19 patients [306]
**Broad-spectrum anti-viral drug**	Nitazoxanide	Broadly amplifying cytoplasmic RNA sensing and type I IFN pathways	Inhibited MERS-CoV infection in LLC-MK2 cells and reduced production of pro-inflammatory cytokines [307]	No reports	Inhibited the viral infection at a low micromolar concentration [258]	Clinical trials are ongoing with limited data
**Combination treatment**	Interferon-α and corticosteroids	Combined anti-viral and anti-inflammatory effects	More rapid resolution of radiographic lung abnormalities and better oxygen saturation levels [232]	No reports	No reports	Lack of randomisation and limited sample size [232]
	Interferon-α and ribavirin	Anti-viral effects and inhibition of viral RNA replication	Highly synergistic effect and more effective than either single treatment [308]	Reduced viral replication and levels of proinflammatory cytokines in infected rhesus macaques [246] The survival rate of patients was improved significantly at 14 days [309]	RBV/IFN-α therapy was not observed to improve clinical outcomes in COVID-19 [310]	Not beneficial for severe and late MERS-CoV infection patients [309]. RBV/IFN-α therapy was associated with a higher probability of hospital stay [310]
	Interferon-β and ribavirin	Anti-viral effects and inhibition of RNA replication	Inhibited viral replication drastically compared to either single treatment in vitro [311]	No mortality reduction and fast viral RNA clearance in patients [312]	Clinical trial combined with lopinavir/ritonavir is ongoing [313]	Clinical data are limited
	Corticosteroids and ribavirin	Combined anti-inflammatory effects and inhibition of viral replication	Evaluated clinical outcomes, including recovered normal production of cytokines for establishing both cell-mediated and humoral immunity in patients [314]	No reports	No reports	Clinical data are limited
	Lopinavir/ritonavir and ribavirin	Combined inhibition of viral replication	Showed stronger suppression on viral load and the adverse clinical outcomes (ARDS or mortality) was significantly lower [234]	Showed rapid viral RNA clearance and patient recovery when combined with IFN-α [315]	Lopinavir/ritonavir in combination with ribavirin and IFNβ-1 b was safe and superior to lopinavir–ritonavir alone in alleviating symptoms and shortening the duration of viral shedding and hospital stay in patients with mild to moderate COVID-19 [316]	Anaemia and fall of haemoglobin in SARS-CoV-1 infection [234] Self-limited nausea and diarrhoea [316]

## Data Availability

Publicly available datasets were analyzed in this study. This data can be found here: WHO|World Health Organization.
